# Long Noncoding RNA ANPODRT Overexpression Protects Nucleus Pulposus Cells from Oxidative Stress and Apoptosis by Activating Keap1-Nrf2 Signaling

**DOI:** 10.1155/2021/6645005

**Published:** 2021-02-02

**Authors:** Liang Kang, Yueyang Tian, Xing Guo, Xu Chu, Yuan Xue

**Affiliations:** ^1^Department of Orthopedics, Tianjin Medical University General Hospital, Tianjin 300052, China; ^2^Tianjin Key Laboratory of Spine and Spinal Cord Injury, Tianjin 300052, China

## Abstract

Oxidative stress and subsequent nucleus pulposus (NP) cell apoptosis are important contributors to the development of intervertebral disc degeneration (IDD). Emerging evidences show that long noncoding RNAs (lncRNAs) play a role in the pathogenesis of IDD. In this study, we investigated the role of lncRNA ANPODRT (anti-NP cell oxidative damage-related transcript) in oxidative stress and apoptosis in human NP cells. We found that ANPODRT was downregulated in degenerative NP tissues and in NP cells treated with tert-butyl hydroperoxide (TBHP, the oxidative stress inducer). ANPODRT overexpression alleviated oxidative stress and apoptosis in NP cells exposed to TBHP, while ANPODRT knockdown exerted opposing effects. Mechanistically, ANPODRT facilitated nuclear factor E2-related factor 2 (Nrf2) accumulation and nuclear translocation and activated its target genes by disrupting the kelch-like ECH-associated protein 1- (Keap1-) Nrf2 association in NP cells. Nrf2 knockdown abolished the antioxidative stress and antiapoptotic effects of ANPODRT in NP cells treated with TBHP. Collectively, our findings suggest that ANPODRT protects NP cells from oxidative stress and apoptosis, at least partially, by activating Nrf2 signaling, implying that ANPODRT may be a potential therapeutic target for IDD.

## 1. Introduction

Low back pain (LBP), one of the most common health issues, is a leading cause of disability worldwide and results in an enormous global economic and public health burden [1]. Intervertebral disc (IVD) degeneration (IDD) and secondary pathomorphological changes in spine structure are widely acknowledged as the major causes of LBP [2, 3]. The IVD consists of three interrelated structures: the nucleus pulposus (NP), the annulus fibrosus, and the cartilaginous endplates. The centrally situated NP allows the IVD to maintain a high water content and withstand different patterns of mechanical stress [4]. Excessive NP cell apoptosis disrupts normal metabolic activity within the NP, thus disrupting the normal IVD structure and physiological function, which is a key contributor to IDD initiation and progression [5, 6].

Oxidative stress results from an imbalance between the production of reactive oxygen species (ROS) and scavenging capacity of the antioxidant system [7]. Oxidative stress leads to the damage of nucleic acids, lipids, and proteins through the production of high levels of ROS, which leads to cytotoxicity. Recent studies have verified the presence of oxidative stress and increased concentrations of oxidation products in degenerated discs [8, 9]. Furthermore, oxidative stress and its induced mitochondrial pathway play an important role in NP cell apoptosis and IDD [10–13]. We and others have previously reported that amelioration of oxidative stress and subsequent NP cell apoptosis has a therapeutic effect on IDD progression [14–16]. Therefore, an understanding of the underlying pathway regulating oxidative stress and subsequent NP cell apoptosis would significantly benefit IDD treatment.

Antioxidant system plays an important role in the occurrence of oxidative stress. The Keap1-Nrf2 signaling cascade is considered a central hub that neutralizes ROS and restores cellular redox balance [17–20]. Under resting conditions, Nrf2 activity is tightly restricted through its interaction with Keap1 in the cytoplasm. Keap1 reportedly serves as a substrate scaffold for Cul3-containing E3 ubiquitin ligase, which can induce ubiquitin-proteasome degradation of Nrf2 [17]. Upon dissociation from Keap1, Nrf2 is translocated into the nucleus, where it initiates the transcription of a battery of antioxidative and cellular defense targets to counteract oxidative stress and modulate redox balance [20]. In a mouse model of IDD, degenerative changes in IVDs in Nrf2-knockout mice were more severe than those in wild-type mice [3]. We previously reported that the activation of the Nrf2 cascade is an effective strategy to protect human disc cells from oxidative damage and prevent IDD progression [14, 15]. Therefore, Nrf2 signal plays an important role in IDD characterized by oxidative stress.

Long noncoding RNAs (lncRNAs) are a large and diverse class of ncRNAs whose transcripts are >200 nt with limited or no protein-coding capacity. Increasing evidence indicates that lncRNAs are implicated in almost all physiological and pathological processes, including IDD [21, 22]. Numerous lncRNAs are dysregulated in IDD. LncRNAs are reportedly upregulated in IDD include RP11-296A18.3 [23], TUG1 [24], HCG18 [25], SNHG1 [26], and NEAT1 [27]. LncRNAs reportedly downregulated in IDD include linc-ADAMTS5 [22], MALAT1 [28], and MAGI2-AS3 [29]. In addition, SNHG6 functions as a promoter of NP cell apoptosis by regulating miR-101-3p [30]. NEAT1 downregulation suppressed advanced glycation end product-induced apoptosis in NP cells [31]. Although several lncRNAs have been studied in IDD, >58,000 lncRNAs have been identified in human cells, compared to 21,000 protein-coding genes in human cells [32]. Therefore, other lncRNAs potentially play a vital role in IDD development, which requires further investigation.

Many lncRNAs dysregulated in IDD have been previously identified through a lncRNA microarray analysis [33]. Among these differentially expressed lncRNAs, lncRNA AC068196.1 was significantly downregulated in IDD. We named lncRNA AC068196.1 as anti-NP cell oxidative damage-related transcript (ANPODRT). This study investigated the role of ANPODRT in oxidative stress and apoptosis in human NP cells. We showed that ANPODRT alleviates oxidative stress and apoptosis in human NP cells, and the activation of the Keap1-Nrf2 signaling cascade is involved in this process. Our results provide novel insights into the mechanism of oxidative stress and apoptosis in NP cells, with therapeutic implications for treating IDD.

## 2. Material and Methods

### 2.1. Tissue Specimens and Cell Culture

This study was approved by the Ethics Committee of Tianjin Medical University General Hospital, and written informed consent was obtained from each donor. Nucleus pulposus specimens were collected from 16 IDD patients (3 males and 2 females, grade II; 2 males and 1 female, grade III; 2 males and 2 females, grade IV; 1 male and 3 females, grade V). Nucleus pulposus cell isolation and culture were carried out as previously described [34]. Five human NP tissues (grade II) were used for NP cell isolation. Briefly, the NP tissue samples were separated, cut into small pieces, and treated with 0.25% trypsin (Gibco) for 30 min and then 0.2% type II collagenase (Invitrogen) for 4 h at 37°C. After isolation, the NP cells were resuspended in DMEM/F12 containing 15% fetal bovine serum (FBS, Gibco) and 1% penicillin-streptomycin and incubated at 37°C in a humidified 5% CO_2_ atmosphere. The culture medium was replaced every three days. When the NP cells grew to 80% confluence, they were detached by trypsinization and passed for expansion. Fluorescently labeled antibodies against NP cell markers (CD24 and KRT18) were used to identify the phenotype of NP cells, as described previously [35]. Cells from the second passage were used in subsequent experiments.

### 2.2. Cell Transfection

The siRNA targeting ANPODRT, Keap1, Nrf2, and appropriate negative controls were obtained from RiboBio. The overexpression plasmids containing ANPODRT and the matched negative control plasmid were obtained from GeneChem. Cell transfections were performed with lipofectamine 2000 (Invitrogen) based on provided instructions.

### 2.3. Cell Viability Assay

According to the manufacturer's instructions, the viability of human NP cells was assessed using a cell counting kit (CCK-8; Dojindo). Briefly, the cells were seeded in 96-well plates and exposed to corresponding treatments. Subsequently, the cells were incubated with 10 *μ*L CCK-8 solution at 37°C for 2 hours. The absorbance at 450 nm was measured using a spectrophotometer (BioTek).

### 2.4. Measurement of ROS and Malondialdehyde (MDA) Level

After corresponding treatment, the ROS and MDA level in human NP cells were measured using kit for ROS (Beyotime) and MDA (Beyotime), respectively, as per manufacturer instructions.

### 2.5. Flow Cytometry

Apoptosis levels in human NP cells from each treatment group were assessed using an Annexin V-APC/7-AAD Apoptosis Detection Kit (Yeasen) as previously described. After labelling, samples were examined using a FACSCalibur flow cytometer (BD Biosciences).

### 2.6. Western Blotting and Coimmunoprecipitation (Co-IP)

After cell treatments, protein samples from NP cells were extracted using commercial kits (Beyotime) according to the manufacturer's instructions. Protein concentrations were determined with a BCA Protein Assay Kit (Beyotime). Equal amounts of protein from each sample were separated using SDS-PAGE and transferred to PVDF membranes. The membranes were blocked with 7.5% nonfat milk for 1 hour and then incubated at 4°C overnight with the following primary antibodies: cytochrome c, cleaved caspase-3, Nrf2, and Keap1. GAPDH and LaminB were used as internal controls. The membranes were subsequently incubated with the respective secondary antibodies at room temperature for 1 hour. Protein bands were visualized by the enhanced chemiluminescence method (Amersham Biosciences) according to the manufacturer's instructions. Band intensities were quantified using the ImageJ software (NIH).

For Co-IP analysis, anti-Keap1 or control IgG was used as the primary antibody, and then the antibody-protein complex was following incubated with Protein A/G PLUS-Agarose. The agarose-antibody-protein complex was collected and then analyzed by Western blotting.

### 2.7. RNA Extraction and Quantitative Real-Time PCR (qRT-PCR)

After treatments of human NP cells in the different groups, total RNA was extracted using a TRIzol reagent (Invitrogen, Carlsbad, CA, USA) and reverse-transcribed using a Transcriptor First Strand cDNA Synthesis Kit (Takara Biotechnology, Otsu, Japan). qRT-PCR was conducted using SYBR Green Kit Master Mix (Applied Biosystems, Foster City, CA, USA), and the products were analyzed using an ABI 7500 Sequencing Detection System, according to the manufacturer's instructions. GAPDH was used as an internal control.

### 2.8. Statistical Analysis

Data are presented as the mean ± standard deviation (SD) of at least three independent experiments and were analyzed using the SPSS version 18.0 software (SPSS Inc, Chicago, IL, USA). Differences between groups were evaluated using Student's *t*-test or one-way analysis of variance (ANOVA) followed by Tukey's test. *p* < 0.05 was considered statistically significant.

## 3. Results

### 3.1. ANPODRT Was Downregulated in Degenerative NP Tissues and TBHP-Stimulated NP Cells

**Figure 1:**
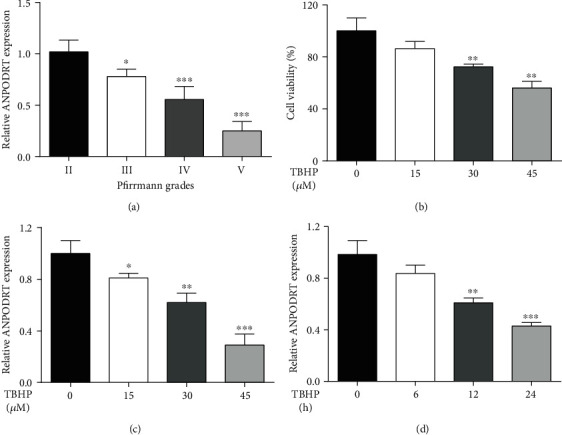
ANPODRT was downregulated in degenerative NP tissues and TBHP-stimulated NP cells. (a) The expression of ANPODRT from NP tissues of different degrees of IDD patients was analyzed by qRT-PCR. ^∗^*p* < 0.05 and ^∗∗∗^*p* < 0.001 versus Pfirrmann grade II group. (b) CCK-8 assay was used to determine the cytotoxic effects of TBHP on human NP cells by treating the cells with various concentrations of TBHP for durations of 24 h. (c) The expression of ANPODRT in human NP cells that treated with different TBHP concentrations for 24 h. (d) The expression of ANPODRT in human NP cells that treated with 45 *μ*M TBHP for different time. Data are represented as the mean ± SD. ^∗^*p* < 0.05, ^∗∗^*p* < 0.01, and ^∗∗∗^*p* < 0.001 versus control group.

ANPODRT is significantly downregulated in degenerative NP tissues. To investigate the association between ANPODRT and IDD, NP tissues of patients with different degrees of degeneration were harvested to determine the ANPODRT expression levels through qRT-PCR. As shown in Figure 1(a), in this study, ANPODRT was downregulated in NP tissues with the degree of disc degeneration. Since oxidative stress is an essential contributor to IDD pathophysiology, TBHP, an exogenous ROS donor, was applied to establish an IDD model in vitro. The CCK-8 assay revealed that TBHP inhibited NP cell viability (Figure 1(b)). Notably, TBHP treatment downregulated ANPODRT in a dose- and time-dependent manner in human NP cells (Figures 1(c) and 1(d)). These results indicate that ANPODRT is potentially associated with IDD.

### 3.2. ANPODRT Overexpression Attenuates TBHP-Induced Oxidative Stress and Apoptosis in Human NP Cells

**Figure 2:**
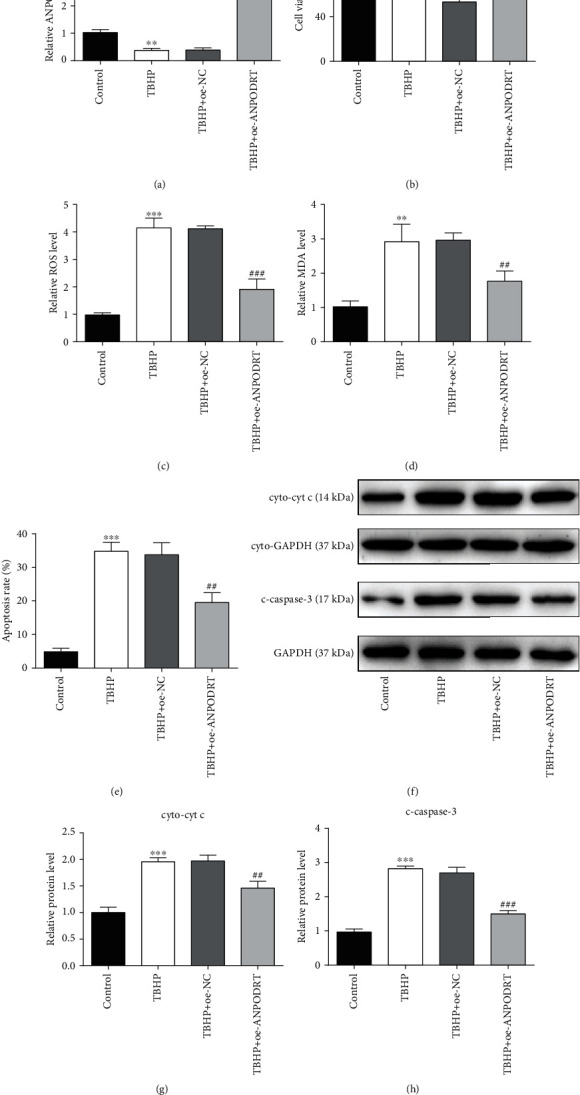
ANPODRT overexpression attenuates TBHP-induced oxidative stress and apoptosis in human NP cells. Human NP cells were transfected with ANPODRT overexpression vector (oe-ANPODRT) or negative control (oe-NC) and then exposed to TBHP. (a) The expression of ANPODRT in human NP cells was detected by qRT-PCR. (b) Cell viability of human NP cells was detected by CCK-8 assay. (c) Intracellular ROS production and (d) MDA level in the human NP cells. (e) Annexin V-APC/7-AAD staining results showing the rate of apoptosis in human NP cells. (f–h) The protein levels of cytoplasmic cytochrome c (cyto-cyt c) and cleaved caspase-3 (c-caspase-3) in the human NP cells were measured by Western blotting. Data are represented as the mean ± SD. ^∗∗^*p* < 0.01 and ^∗∗∗^*p* < 0.001 versus control group; ^##^*p* < 0.01 and ^###^*p* < 0.001 versus TBHP+oe-NC group.

To determine whether ANPODRT plays a role in the TBHP-induced IDD model in vitro, we used an ANPODRT expression vector to overexpress ANPODRT in TBHP-treated human NP cells. qRT-PCR confirmed the overexpression efficiency (Figure 2(a)). Oxidative stress and its induced mitochondrial pathway-mediated apoptosis of NP cells play an important role in IDD pathogenesis. Therefore, indicators of oxidative stress and mitochondrial apoptotic pathway were subsequently assessed. The CCK-8 assay revealed that ANPODRT overexpression inhibited the reduction in NP cell viability induced by TBHP (Figure 2(b)). Furthermore, ROS and MDA levels were determined to evaluate oxidative stress levels in human NP cells. As shown in Figures 2(c) and 2(d), TBHP treatment elevated the ROS and MDA levels, which was suppressed by ANPODRT overexpression. Moreover, flow cytometry revealed that ANPODRT overexpression alleviated the TBHP-induced increase in NP cell apoptosis (Figure 2(e)). Mitochondrial cytochrome c (cyt c) release and caspase-3 activation induced by cyt c are important features of mitochondrial pathway-mediated apoptosis. We found that protein levels of cytoplasmic cyt c and cleaved caspase-3 were significantly increased in human NP cells treated with TBHP, and these TBHP-induced alterations were prevented through ANPODRT overexpression (Figures 2(f)–2(h)).

### 3.3. ANPODRT Knockdown Aggravates TBHP-Induced Human NP Cell Injury

**Figure 3:**
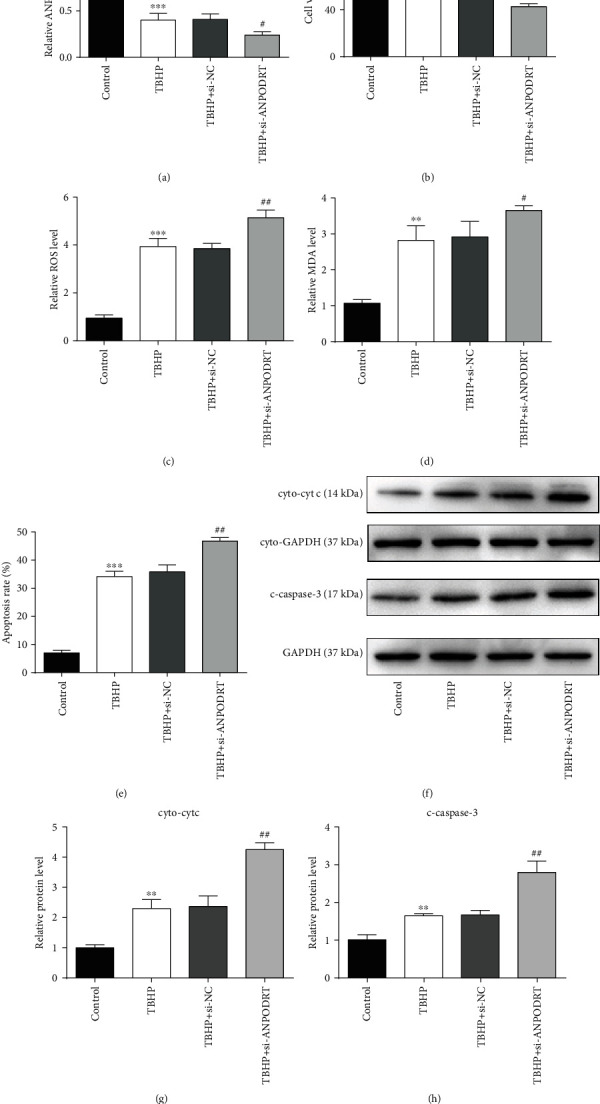
ANPODRT knockdown aggravates TBHP-induced human NP cell injury. Human NP cells were transfected with short interfering RNA (siRNA) against ANPODRT (si-ANPODRT) or negative control (si-NC) and then exposed to TBHP. (a) The expression of ANPODRT in human NP cells was detected by qRT-PCR. (b) Cell viability of human NP cells was detected by CCK-8 assay. (c) Intracellular ROS production and (d) MDA level in the human NP cells. (e) Annexin V-APC/7-AAD staining results showing the rate of apoptosis in human NP cells. (f–h) The protein levels of cytoplasmic cytochrome c (cyto-cyt c) and cleaved caspase-3 (c-caspase-3) in the human NP cells were measured by Western blotting. Data are represented as the mean ± SD. ^∗∗^*p* < 0.01 and ^∗∗∗^*p* < 0.001 versus control group; ^#^*p* < 0.05, ^##^*p* < 0.01, and ^###^*p* < 0.001 versus TBHP+si-NC group.

To investigate the role of ANPODRT in the TBHP-induced IDD model in vitro, ANPODRT expression was knocked down using siRNA in TBHP-treated human NP cells. As shown in Figure 3(a), qRT-PCR analysis revealed that ANPODRT downregulation induced by TBHP in human NP cells was aggravated through transfection with si-ANPODRT. Functional studies revealed that contrary to ANPODRT overexpression, ANPODRT knockdown further exacerbated TBHP-induced oxidative stress and apoptosis in human NP cells, as manifested by a reduction in cell viability (Figure 3(b)) and an increase in ROS accumulation (Figure 3(c)), MDA production (Figure 3(d)), apoptosis rate (Figure 3(e)), cytoplasmic cyt c level (Figures 3(f) and 3(g)), and cleaved caspase-3 level (Figures 3(f) and 3(h)).

### 3.4. ANPODRT Activates Nrf2 Signaling in Human NP Cells

**Figure 4:**
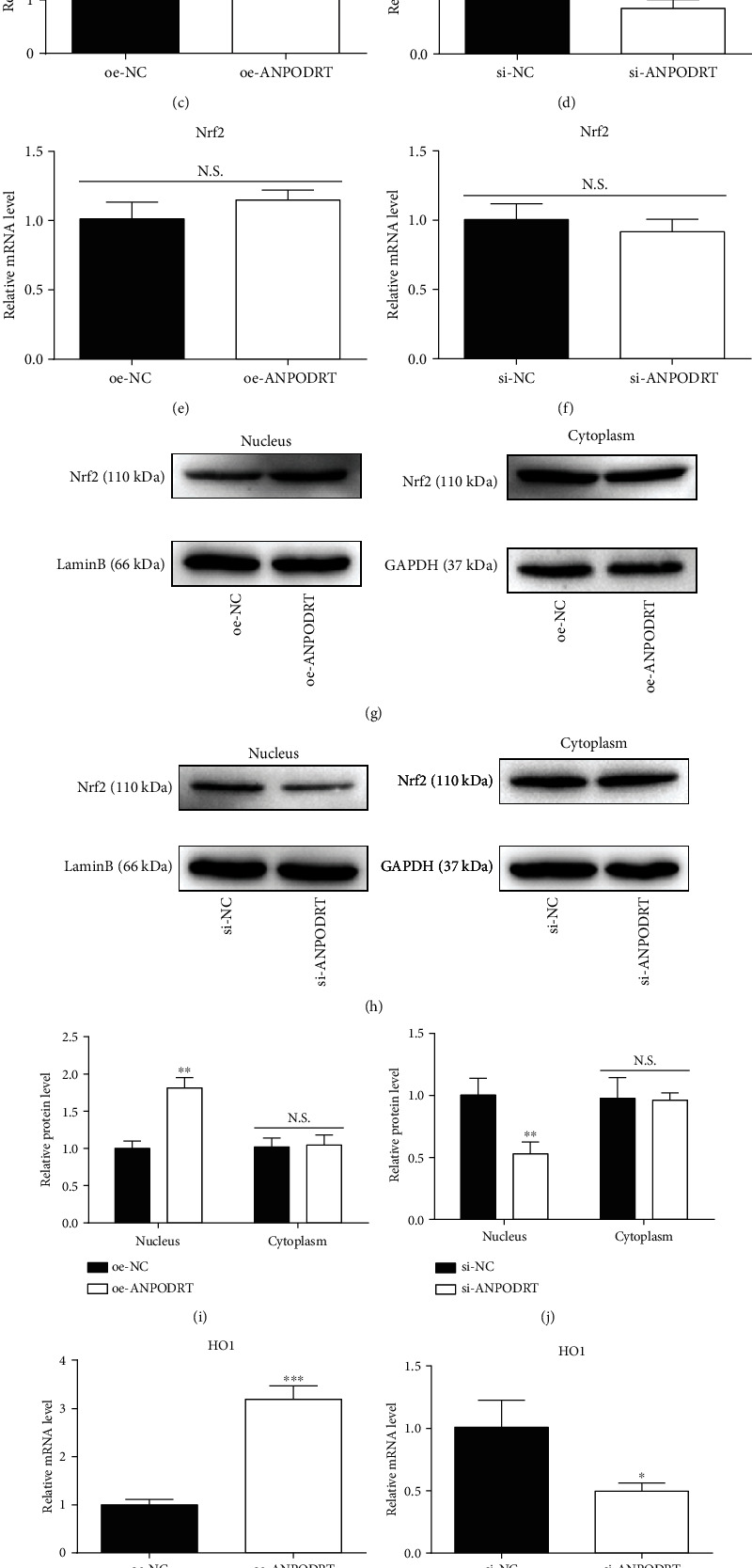
ANPODRT activates Nrf2 signaling in human NP cells. (a–d) The protein expression of Nrf2 in human NP cells transfected with oe-ANPODRT, si-ANPODRT, or negative control was measured by Western blotting. (e, f) The mRNA expression of Nrf2 in human NP cells transfected with oe-ANPODRT, si-ANPODRT, or negative control was measured by qRT-PCR. (g–j) The protein expression of nucleus Nrf2 and cytoplasm Nrf2 was measured by Western blotting. (k–n) The mRNA expression of HO1 and NQO1 in human NP cells transfected with oe-ANPODRT, si-ANPODRT, or negative control was measured by qRT-PCR. Data are represented as the mean ± SD. ^∗^*p* < 0.05, ^∗∗^*p* < 0.01, and ^∗∗∗^*p* < 0.001 versus si-NC/oe-NC group; N.S.: not significant.

Nrf2 is a key regulator of the cellular antioxidative defense system. The activation of Nrf2 signaling can efficiently protect human intervertebral disc cells from oxidative stress. To further investigate the mechanism underlying ANPODRT-mediated inhibition of oxidative injury in human NP cells, we examined whether ANPODRT expression affects Nrf2 signaling. Our results show that ANPODRT overexpression significantly increased the protein level of Nrf2 in human NP cells. In contrast, ANPODRT knockdown markedly downregulated Nrf2 protein (Figures 4(a)–4(d)). Remarkably, Nrf2 mRNA levels were not altered through ANPODRT overexpression or knockdown (Figures 4(e) and 4(f)). These results indicate that ANPODRT positively regulated Nrf2 accumulation, which was not observed at the transcriptional level. Furthermore, we investigated the regulation of ANPODRT on the intracellular distribution of Nrf2 protein. As shown in Figures 4(g)–4(j), ANPODRT overexpression increased Nrf2 nuclear translocation, while ANPODRT knockdown decreased it. Nuclear Nrf2 can bind to the antioxidant response element and then activate its target genes to counteract oxidative stress and modulate redox status balance. To evaluate the transcriptional activity of Nrf2, mRNA expression of well-established Nrf2-dependent genes, including HO1 and NQO1, were detected through qRT-PCR. We found that HO1 and NQO1 mRNA levels were increased after ANPODRT overexpression in human NP cells, while those of HO1 and NQO1 were decreased after ANPODRT knockdown (Figures 4(k)–4(n)). Collectively, these results suggest that ANPODRT promotes accumulation, nuclear translocation, and Nrf2 activation in human NP cells.

### 3.5. ANPODRT Disrupts the Keap1-Nrf2 Complex Formation in Human NP Cells

**Figure 5:**
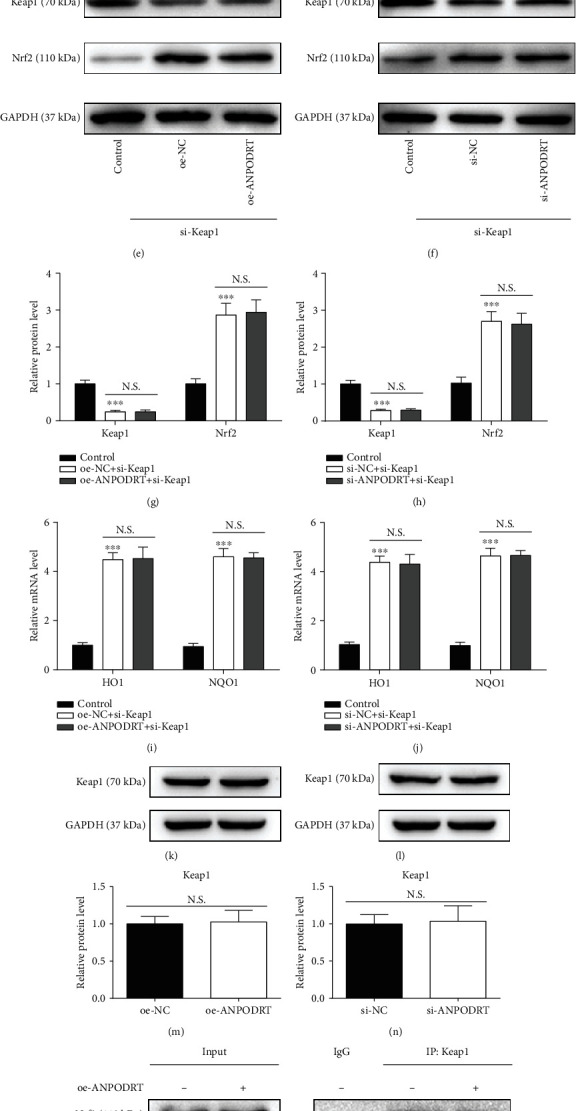
ANPODRT disrupts the Keap1-Nrf2 complex formation in human NP cells. (a–d) Human NP cells were transfected with oe-ANPODRT, si-ANPODRT, or negative control followed by MG132 treatment. The protein level of Nrf2 in human NP cells was measured. (e–h) The protein level of Keap1 and Nrf2 in human NP cells transfected using oe-ANPODRT/si-ANPODRT and si-Keap1. (i, j) The mRNA level of HO1 and NQO1 in human NP cells transfected using oe-ANPODRT/si-ANPODRT and si-Keap1. (k–n) The protein level of Keap1 in human NP cells transfected with oe-ANPODRT, si-ANPODRT, or negative control. (o, p) After oe-ANPODRT/si-ANPODRT transfection, the interaction between Keap1 and Nrf2 was determined by Co-IP assay. Data are represented as the mean ± SD. ^∗∗∗^*p* < 0.001 versus control group; N.S.: not significant.

Furthermore, we investigated how ANPODRT regulates Nrf2 signaling in human NP cells. Nrf2 can not only be captured by Keap1, but also undergo Keap1-mediated ubiquitination and subsequent proteasomal degradation. Consequently, the proteasome inhibitor MG132 increased Nrf2 protein levels (Figures 5(a)–5(d)). Notably, MG132 also abolished the regulation of ANPODRT overexpression or knockdown on Nrf2 expression (Figures 5(a)–5(d)). The present data indicate that proteasomal Nrf2 degradation is involved in Nrf2 regulation by ANPODRT. Furthermore, Nrf2 degradation may be induced in a Keap1-independent manner. Therefore, we investigated whether Keap1 is involved in ANPODRT-induced Nrf2 accumulation in human NP cells. These results indicate that in Keap1-knockdown human NP cells, ANPODRT overexpression and knockdown were both ineffective in regulating Nrf2 accumulation (Figures 5(e)–5(h)) and Nrf2-dependent gene (HO1 and NQO1) expression (Figures 5(i) and 5(j)), suggesting that ANPODRT regulates Nrf2 accumulation primarily by interfering with Keap1. Nonetheless, Keap1 expression was not affected through ANPODRT expression (Figures 5(k)–5(n)). Therefore, we speculated that ANPODRT potentially disrupts the Keap1-Nrf2 association. To investigate this possibility, we analyzed the association between Keap1 and Nrf2 in human NP cells after ANPODRT overexpression or knockdown. Co-IP assays revealed that ANPODRT overexpression reduced the association between Keap1 and Nrf2 in human NP cells (Figure 5(o)). In contrast, the association between Keap1 and Nrf2 was increased upon ANPODRT knockdown (Figure 5(p)). Together, these findings suggest that ANPODRT induces the dissociation of the Keap1-Nrf2 complex, thus activating the Nrf2 cascade in human NP cells.

### 3.6. Nrf2 Activation Is Required for ANPODRT-Induced Human NP Cell Protection against Oxidative Injury

**Figure 6:**
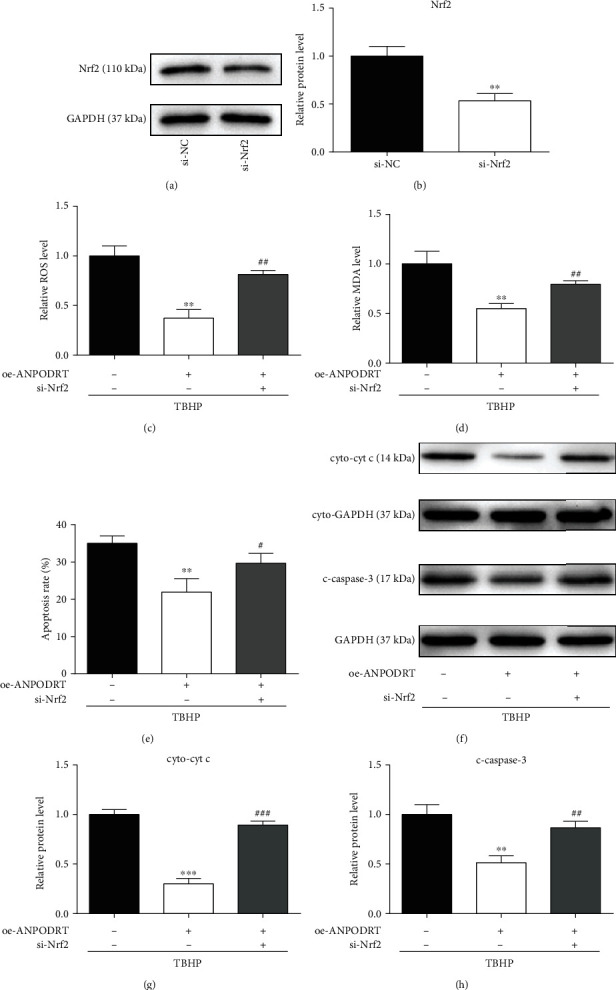
Nrf2 activation is required for ANPODRT-induced human NP cell protection against oxidative injury. (a, b) The protein level of Nrf2 in human NP cells transfected with si-Nrf2 or negative control. ^∗∗^*p* < 0.01 versus si-NC group. (c–h) Human NP cells were transfected with oe-ANPODRT and si-Nrf2 followed by TBHP treatment. (c) Intracellular ROS production and (d) MDA level in the human NP cells. (e) Annexin V-APC/7-AAD staining results showing the rate of apoptosis in human NP cells. (f–h) The protein levels of cytoplasmic cytochrome c (cyto-cyt c) and cleaved caspase-3 (c-caspase-3) in the human NP cells were measured by Western blotting. Data are represented as the mean ± SD. ^∗∗^*p* < 0.01 and ^∗∗∗^*p* < 0.001 versus TBHP treatment only group; ^#^*p* < 0.05, ^##^*p* < 0.01, and ^###^*p* < 0.001 versus TBHP+oe-ANPODRT group.

The present results show that ANPODRT overexpression activates the Nrf2 cascade and protects human NP cells from oxidative injury. To confirm whether Nrf2 mediates the protective effects of ANPODRT, rescue experiments were performed through siRNA-mediated Nrf2 silencing. Western blotting confirmed the Nrf2 knockdown efficiency (Figures 6(a) and 6(b)). Importantly, the protection of ANPODRT overexpression against TBHP-induced oxidative stress and apoptosis in human NP cells was reversed through Nrf2 knockdown, as indicated through the results of ROS levels (Figure 6(c)), MDA production (Figure 6(d)), apoptosis rate (Figure 6(e)), cytoplasmic cyt c levels (Figures 6(f) and 6(g)), and cleaved caspase-3 levels (Figures 6(f) and 6(h)). Together, these results indicate that the effects of ANPODRT overexpression-induced antioxidative injury depend on the activation of the Nrf2 cascade in human NP cells.

## 4. Discussion

Although numerous studies have reported the important roles and clinical significance of lncRNAs in various diseases, the functional roles of most of these molecules are unknown, particularly in the context of IDD [36–38]. In this study, we investigated the expression and function of ANPODRT in IDD. We observed a decreasing trend for ANPODRT expression during IDD pathogenesis, and the well-recognized oxidative stress inducer TBHP downregulated ANPODRT in human NP cells in a dose- and time-dependent manner. Gain-of-function and loss-of-function assays indicated that ANPODRT overexpression suppressed oxidative stress and apoptosis in TBHP-treated human NP cells, while ANPODRT knockdown exerted opposing effects. Mechanistically, these results show that ANPODRT disrupted the Keap1-Nrf2 association, inducing Nrf2 accumulation and nuclear translocation and the expression of Nrf2 target genes in human NP cells. Nrf2 knockdown abolished lncRNA-induced antioxidative stress and antiapoptotic effects in TBHP-treated human NP cells. These data indicate that ANPODRT inhibits oxidative stress and apoptosis in human NP cells, thus highlighting it as a potential target for IDD treatment.

Accumulating evidence indicates that degenerated IVDs exhibit increased ROS levels, which contributes to IDD pathogenesis. Furthermore, TBHP has several advantages over H_2_O_2_, such as high stability and gradual release. Accordingly, previous reports support the in vitro TBHP-induced IDD model used herein [39, 40]. Apoptosis can be initiated by the extrinsic death receptor pathway and the intrinsic mitochondrial apoptosis pathway. The latter has been confirmed to be deeply involved in the increased NP cell death in IDD pathogenesis [16, 39]. Oxidative stress-induced dysfunctional mitochondria can release proapoptotic proteins to form the apoptosome and activate caspase cascades for NP cell apoptosis. Cytoplasmic translocation of mitochondrial cyt c and caspase-3 activation induced by cyt c are characteristics of NP cell apoptosis through the mitochondrial pathway [41]. Several lncRNAs reportedly participate in oxidative stress and the mitochondrial apoptosis pathway. For example, Niu et al. reported that lncRNA Oip5-as1 suppresses microRNA-29a, thus activating the SIRT1/AMPK/PGC1*α*pathway, which attenuates oxidative stress and mitochondria-mediated apoptosis during myocardial ischemia/reperfusion injury [42]. Li et al. reported that lncRNA H19 overexpression attenuates H_2_O_2_-induced cardiomyocyte injury by suppressing microRNA-877-3p/Bcl-2 pathway-mediated mitochondrial apoptosis [43]. However, the role of ANPODRT in oxidative stress and its induction of the mitochondrial apoptosis pathway in human NP cells remain unknown. Therefore, we performed a functional analysis of ANPODRT to determine whether it is involved in oxidative stress and human NP cell apoptosis during IDD. The present results show that ANPODRT was downregulated during IDD pathogenesis, and oxidative stress induced by TBHP downregulated ANPODRT. Notably, ANPODRT overexpression attenuated the accumulation of ROS and MDA, suppressed the upregulation of cytoplasmic-cyto-c and cleaved caspase-3, and inhibited the elevation of the apoptotic rate in human NP cells stimulated with TBHP. These effects were reversed through ANPODRT inhibition. These findings suggest that ANPODRT inhibits oxidative stress and apoptosis in human NP cells.

Mechanistically, considering that ANPODRT inhibits oxidative stress in human NP cells and that the Keap1-Nrf2 complex is an important regulator of oxidative stress, we investigated whether the Keap1-Nrf2 complex is involved in the ANPODRT-mediated protective effects in TBHP-treated human NP cells. Lv et al. reported that eriodictyol-induced retinal ganglion cell protection against high glucose-induced oxidative stress and apoptosis is associated with the activation of Nrf2 signaling [44]. Moreover, along with direct Nrf2 activation, the disruption of the Keap1-Nrf2 interaction has been widely studied. Certain internal disruptors including p62 [45], iASPP [17], and lncRNA-Sox2OT [46] reportedly interfere with the Keap1-Nrf2 complex, thus decreasing the Keap1-Nrf2 interaction and subsequently activating Nrf2 signaling, ultimately affecting the antioxidative status of the cell. Lu et al. reported that CPUY192018, a potent inhibitor of the Keap1-Nrf2 interaction, alleviates renal inflammation in mice by restricting oxidative stress and NF-*κ*B activation [19]. The present results show that ANPODRT induced Keap1-Nrf2 disassociation, leading to Nrf2 protein accumulation and nuclear translocation and the expression of Nrf2 targets, including HO1 and NQO1, in human NP cells. Furthermore, Nrf2 knockdown almost obliterated ANPODRT-induced protection of human NP cells against oxidative stress and apoptosis. These results suggest that Nrf2 activation is required for ANPODRT-induced cytoprotective effects in TBHP-treated human NP cells.

There are several difficulties in isolating and culturing NP cells. For the isolation of NP cells, because excessive enzyme digestion will destroy the cells, we cut the NP tissue into small pieces as much as possible, so that the tissue can be in better contact with the enzyme, thus shortening the enzyme digestion time. In the process of primary cell culture, we use penicillin and streptomycin to solve the problem that primary cell culture is easy to be contaminated.

3D culture and hypoxic environment are two aspects that cannot be ignored in the study of IDD. The physiological properties of the intervertebral disc are linked to the structure of its extracellular matrix (ECM) [5]. The ECM in healthy intervertebral disc is a 3D network of natural nanoscale fibers. NP cells in vivo are surrounded by ECM. NP cells can easily lose their phenotype characteristics in monolayer growth [47]. In addition, there are steep gradients in oxygen concentrations across the avascular intervertebral disc, with O_2_ falling to as low as 1% in the center of a large disc [48]. A low oxygen tension environment plays a vital role in maintaining intervertebral disc physiological function, including matrix synthesis and even cell metabolism [49]. Studies have shown that a physiological oxygen of 1% appears to promote the best NP phenotype for bovine intervertebral disc cells in alginate [48]. In addition, Pei et al. have reported that low oxygen tension (5%) enhanced the ECM synthesis of porcine NP cells in a pellet culture system [50]. Therefore, the application of 3D culture in hypoxia can better reflect the actual environment of intervertebral disc. The role of lncRNA in IDD needs more investigations in 3D culture under hypoxia in the future.

Another limitation of this study was that our experiments were performed in vitro, and in vitro results may not directly reflect the clinical settings. Thus, in vivo experiments are needed to confirm the role of ANPODRT in IDD and to increase our understanding of the molecular mechanisms underlying this effect.

In summary, this study shows that ANPODRT protects human NP cells from oxidative stress and apoptosis, at least partially, by interfering with Keap1-Nrf2 association and activating Nrf2-mediated antioxidant system. Our findings further the current understanding of the pathomechanism of IDD and suggest that ANPODRT is a potentially promising therapeutic target for IDD.

## Data Availability

The data used to support the findings of this study are included within the article.

## Abbreviations

LBP: Low back pain
IDD: Intervertebral disc degeneration
NP: Nucleus pulposus
ROS: Reactive oxygen species
LncRNAs: Long noncoding RNAs
MDA: Malondialdehyde
Nrf2: Nuclear factor E2-related factor 2
Keap1: Kelch-like ECH-associated protein 1
TBHP: Tert-butyl hydroperoxide.
